# The defining DNA methylation signature of Floating-Harbor Syndrome

**DOI:** 10.1038/srep38803

**Published:** 2016-12-09

**Authors:** Rebecca L. Hood, Laila C. Schenkel, Sarah M. Nikkel, Peter J. Ainsworth, Guillaume Pare, Kym M. Boycott, Dennis E. Bulman, Bekim Sadikovic

**Affiliations:** 1 Department of Biochemistry, Microbiology and Immunology, University of Ottawa, 451 Smyth Road, Ottawa, K1H 8M5, Canada; 2 Children’s Hospital of Eastern Ontario Research Institute, University of Ottawa, 401 Smyth Road, Ottawa, K1H 5B2, Canada; 3 Department of Pathology and Laboratory Medicine, Western University, 1151 Richmond Street, London, N6A 3K7, Canada; 4 Provincial Medical Genetics Program, BC Women’s Hospital and Health Centre, 4500 Oak Street, Vancouver, V6H 3N1, Canada; 5 Lawson Research Institute, London Health Sciences Centre, 750 Base Line Road East, London, N6A 5W9, Canada; 6 Molecular Genetics Laboratory, London Health Sciences Centre, 800 Commissioners Road East, London, N6A 5W9, Canada; 7 Children’s Health Research Institute, 800 Commissioners Road East, London, N6A 5W9, Canada; 8 Department of Pathology and Molecular Medicine and Department of Clinical Epidemiology and Biostatistics, McMaster University, 1280 Main Street West, Hamilton, L8S 4L8, Canada; 9 Department of Pediatrics, University of Ottawa, 451 Smyth Road, Ottawa, K1H 8L1, Canada

## Abstract

Floating-Harbor syndrome (FHS) is an autosomal dominant genetic condition characterized by short stature, delayed osseous maturation, expressive language impairment, and unique facial dysmorphology. We previously identified mutations in the chromatin remodeling protein SRCAP (SNF2-related CBP Activator Protein) as the cause of FHS. SRCAP has multiple roles in chromatin and transcriptional regulation; however, specific epigenetic consequences of SRCAP mutations remain to be described. Using high resolution genome-wide DNA methylation analysis, we identified a unique and highly specific DNA methylation “epi-signature” in the peripheral blood of individuals with FHS. Both hyper and hypomethylated loci are distributed across the genome, preferentially occurring in CpG islands. Clonal bisulfite sequencing of two hypermethylated (*FIGN* and *STPG2*) and two hypomethylated (*MYO1F* and *RASIP1*) genes confirmed these findings. The identification of a unique methylation signature in FHS provides further insight into the biological function of SRCAP and provides a unique biomarker for this disorder.

Floating-Harbor syndrome (FHS; MIM 136140) is a rare autosomal dominant genetic disorder characterized by short stature, delayed osseous maturation, expressive language impairment, and facial dysmorphology^1^,^2^,^3^,^4^. The facial features characteristic of FHS include: a triangular-shaped face, prominent nose, short philtrum, and a wide flat mouth with a thin upper lip. Individuals with FHS typically exhibit language deficits and some level of learning or intellectual disability. FHS usually occurs sporadically; however, a few autosomal dominant parent-child transmissions have been reported^4^,^5^,^6^,^7^. In 2012, we identified heterozygous truncating mutations in the final exon of *SRCAP* (SNF2-related CBP Activator Protein) as the genetic cause underlying FHS^8^. This report was followed by a more in-depth clinical analysis of a large cohort of 52 affected individuals, which better defined both the mutation and the clinical spectrum of FHS^9^. *SRCAP* encodes a large SWI/SNF-type chromatin remodeling ATPase, which was first identified in a yeast two-hybrid screen for CREB-binding protein (CREBBP) interaction partners^10^. Mutations in *CREBBP*, or its homolog, *p300*, are known to cause Rubinstein-Taybi syndrome, another short stature disorder that shares some features with FHS^11^,^12^. Multiple coactivator roles have been described for SRCAP, in CREB and CREBBP-mediated, nuclear (steroid) hormone receptor, and Notch signaling pathways^10^,^13^,^14^. SRCAP has also been shown to immuno-precipitate as part of a large chromatin remodeling complex involved in the ATP-dependent displacement of the histone variant H2A by H2A.Z^15^,^16^. Additionally, the SRCAP-complex is known to function as a regulator of DNA damage and double strand break repair^17^. While many roles for SRCAP have been described, the downstream impact of disease-causing mutations remains largely unknown.

DNA methylation, the addition of a methyl (CH3) group typically to a cytosine residue within CpG dinucleotides, is the most comprehensively described form of epigenetic modification. These epigenetic changes have an essential role in many nuclear functions, and in particular, transcriptional regulation and regulation of chromatin structure. In general, promoter regions with unmethylated CpGs are associated with a transcriptionally permissive chromatin state, whereas methylated CpG islands (high density CpG regions observed at approximately 50% of gene promoters) are associated with transcriptional repression. As such, the fine regulation of methylation constitutes an extra layer of control over gene expression.

Several genes, such as the DNA methyltransferases *DNMT1, DNMT3A*, and *DNMT3B*, have been linked with the regulation of methylation status through interaction with histone deacetylases^18^,^19^. Chromatin remodeling proteins, including two members of the same SNF2-ATPase chromatin family as SRCAP, Lymphoid Specific Helicase (HELLS) and X-linked alpha thalassemia/mental retardation (ATRX), have also been shown to impact methylation status^20^,^21^,^22^,^23^. It is therefore possible that other chromatin remodeling proteins, such as SRCAP, may also impact methylation status. In this case, we hypothesized that the truncating mutations of *SRCAP* seen in FHS could cause differential methylation, and that these differences may provide insight into the pathogenesis of this disorder. We therefore set out to determine if individuals with FHS have a unique DNA methylation epi-signature in their peripheral blood.

## Results

### Differential methylation attributed to mutations in SRCAP

**Figure 1:**
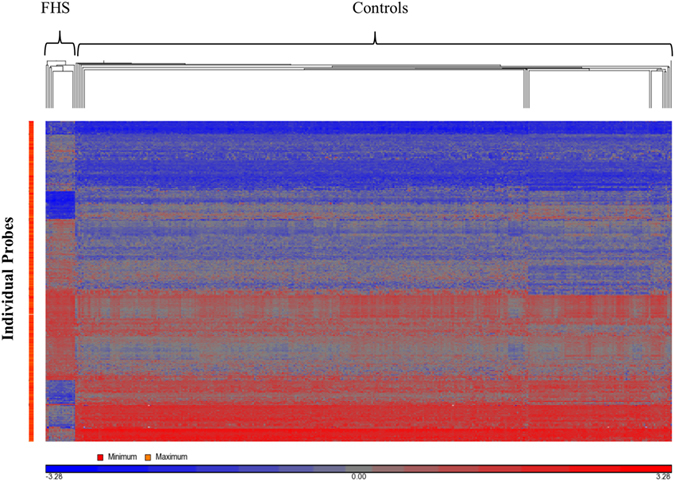
Euclidean Hierarchical Cluster Analysis. Hierarchical clustering of probes differentially methylated between FHS and controls demonstrating marked asymmetry of the 2 groups. Cases are represented in the columns and significant probes (p < 0.01) in the rows.

The methylation array data identified a unique methylation profile specific to FHS individuals. Within the FHS cohort of 18 affected individuals, methylation differences with respect to the particular *SRCAP* mutation were not observed. Additionally, there were no gender-specific global methylation differences or sex chromosome methylation changes found within the FHS cohort. Hierarchical clustering of significant probes (p < 0.01, F > 50, Estimate > 15%) clearly demonstrated a unique methylation profile and sub-clustering for these patients compared with our large laboratory reference cohort (Fig. 1). Overall, a higher frequency of hypermethylation was observed in individuals with FHS, regardless of genomic location and CpG island proximity (Supplementary Fig. 1). A comprehensive list of differentially methylated regions shows 116 loci, 31 of which are hypomethylated and 85 are hypermethylated, with 73 of the 116 loci overlapping CpG islands and 8 overlapping CpG shore (Supplementary Table 1 and 2). The 116 DMRs represent regions with decreased cut-off criteria (methylation difference >15%), and include 28 regions (Table 1) where the more restrictive criteria were used (methylation difference >20%). Of the 28 identified FHS-specific methylation regions: 19 regions were found to have significantly increased methylation (20.01–32.23% higher methylation estimates) in the FHS samples compared to controls, and 9 regions were found to have significantly decreased methylation (20.17–37.25% lower methylation estimates). The majority of these regions were located within genes (n = 17), including 7 in promoters, 6 intragenic and 4 in intronic regions (Table 2). Although only 1/3 of the array probes map to CpG islands, approximately 2/3 (19 of the 28) of the identified regions with differential methylation correspond to locations within CpG islands. Specifically, of these 19 regions, 10 were hypermethylated while 9 were hypomethylated, the latter representing all of the hypomethylated regions identified in the set of 28 and demonstrating a relative increase in the proportion of hypomethylated regions within CpG islands as compared to non-CpG islands for FHS individuals (Supplementary Fig. 1).

### DNA methylation age and cell counts estimation

Average age acceleration was used to determine whether the DNA methylation age of a FHS individuals is consistently higher (or lower) than expected (as in controls). We observed that the average age acceleration did not significantly differ between FHS patients and controls (1.48 ±12 and 2.66 ±3.4, respectively). In addition, the cell type estimation showed no significant differences of blood cell composition between FHS patients and controls (Supplementary Fig. 2). Taken together these results indicate that changes in DNA methylation observed in the FHS cohort cannot be attributed to differences in DNA methylation age and/or blood cell type.

### Validation of methylation assay

**Figure 2:**
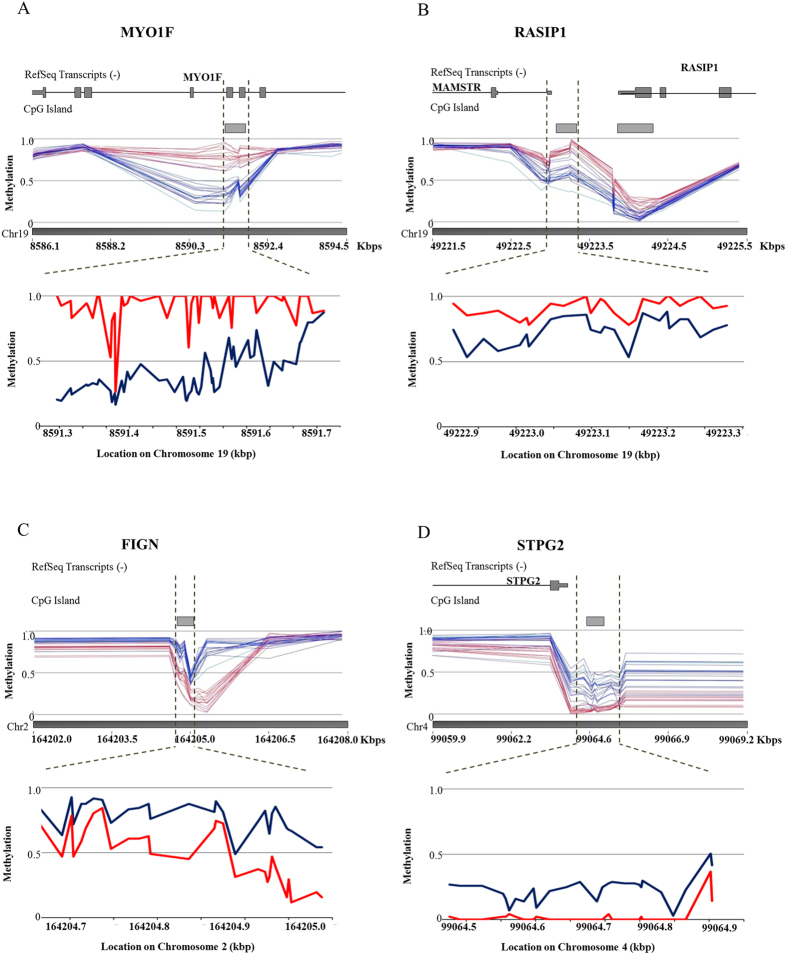
DNA methylation profiles in FHS. Methylation level from 0 (not methylated) to 1 (100% methylated) is shown across regions with significantly altered methylation in FHS: hypermethylated regions (**a**) *MYO1F* and (**b**) *RASIP1*; and hypomethylated regions (**c**) *FIGN* and (**d**) *STPG2*. RefSeq genes and CpG islands tracks are annotated on top of the figures. The top image corresponds to methylation array data visualized using Genomic Browser Viewer (Partek). Red lines correspond to representative control sample data. Blue lines correspond to FHS individual data. The bottom image corresponds to average methylation based on bisulfite sequencing data. The red and blue lines indicate the average methylation for control and FHS patient sample, respectively. Dotted lines correlate chromosome location between top (array generated) and bottom (bisulfite sequence generated) images.

Amongst the 28 regions found to be differentially methylated in FHS, two hypermethylated (*FIGN* and *STPG2*) and two hypomethylated (*MYO1F* and *RASIP1*) regions were selected based on robust methylation differences and statistical significance for comparative analysis of array data to bisulfite sequencing. Methylation array showed a mean of 32.23% and 23.49% hypermethylation in FHS individuals at the *FIGN* and *STPG2* gene loci, respectively (Table 1). Conversely, *MYO1F* and *RASIP1* genes loci showed a mean of 37.25% and 20.17% hypomethylation in the FHS cohort relative to the control cohort (Table 1). These loci were well represented on the array, with multiple probes spanning each of the respective differentially methylated regions. Methylation profiles showed consistent hypermethylation in FHS individuals as compared to controls for both *FIGN* and *STPG2* regions across 7 and 9 probes respectively, and consistent hypomethylation for *MYO1F* and *RASIP1* regions across 4 and 12 probes, respectively (Fig. 2). In addition, samples from the individuals with FHS had a higher average methylation level over the *FIGN* and *STPG2* regions and a lower average methylation level over the *MYO1F* and *RASIP1* regions as compared with controls (Supplementary Fig. 3).

**Figure 3:**
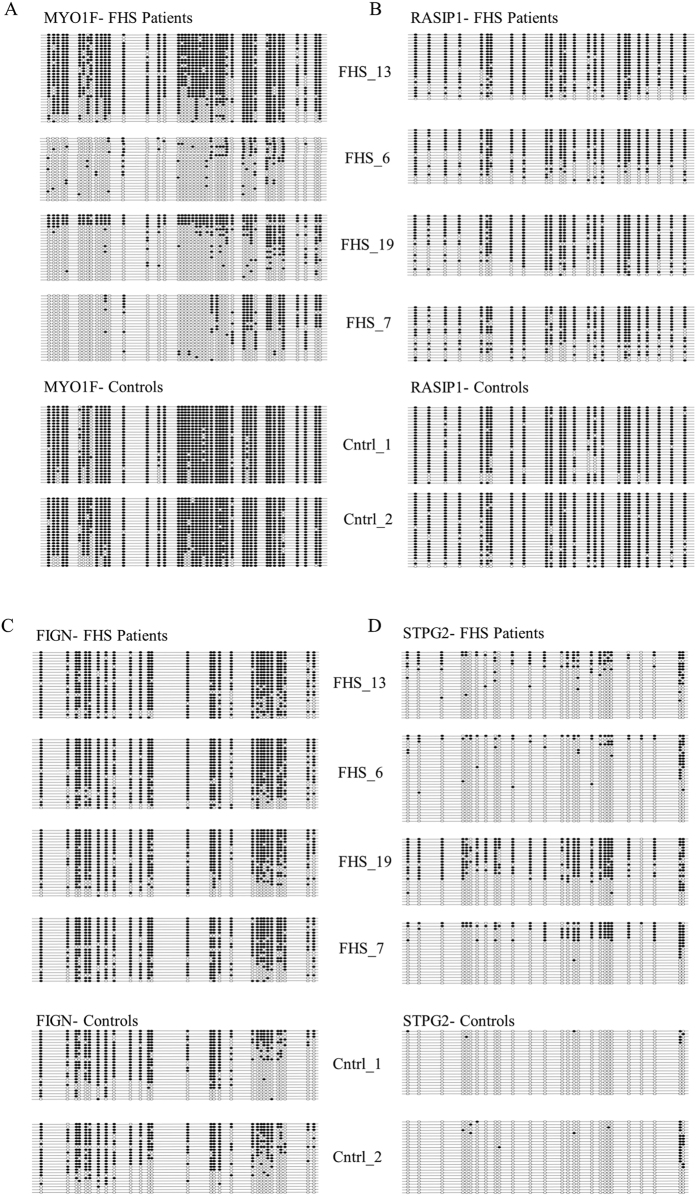
Methylation string diagrams of significantly altered regions in FHS individuals compared to controls. String diagrams indicating methylation status across regions with significantly altered methylation in FHS: hypermethylated regions (**a**) *MYO1F* and (**b**) *RASIP1*, and hypomethylated regions (**c**) *FIGN* and (**d**) *STPG2*; for four FHS individuals (top) and two gender matched control samples (bottom). Each dot on the string indicates a CpG sequence, and potential site for methylation. Black dots indicate the CpG is methylated. Open dots indicate the CpG is un-methylated.

Bisulfite sequencing analyses were performed to technically confirm the FHS-specific methylation profile by examining methylation status across *FIGN, STPG2, MYO1F,* and *RASIP1* regions (Fig. 3). Bisulfite sequencing analysis across the *MYO1F* and *RASIP1* loci included 45 and 25 CpG sites, respectively. Consistent with the array findings, the average degree of methylation for FHS individuals across the *MYO1F* and *RASIP1* regions determined by bisulfite sequencing was correspondingly lower than for controls (Fig. 2a,b). Additionally, the average percent methylation for *MYO1F* in FHS individuals was 37% compared to 92% in controls (Fig. 3a). For *RASIP1* the average percent methylation in FHS individuals versus controls was 73% and 91%, respectively (Fig. 3b). The bisulfite data for these two regions supported the methylation array results, confirming that these two regions are hypomethylated in FHS individuals. For *FIGN* and *STPG2* regions, 29 and 27 CpG sites were examined, respectively. The bisulfite data demonstrated hypermethylation in FHS individuals versus controls (77% versus 49% for *FIGN*, and 24% versus 3% for *STPG2*; Fig. 3c,d). The bisulfite data for the *FIGN* and *STPG2* regions also confirm similar levels of hypermethylation in FHS patients relative to the microarray findings (Fig. 2c,d).

The results of the bisulfite data, which included one independent FHS patient that was not included in the microarray discovery cohort, corroborate the methylation data, confirm the existence of an epigenetic signature associated with FHS, and support the diagnostic utility of such an array-based approach.

### Pathway analysis

Pathway analysis, performed using the list of 116 differentially methylated genes (>15%; Supplementary Table 1) identified significantly enriched gene groups involved in a number of biological processes. More specifically, an over-representation of genes was found to be involved in synaptic transmission and the neurological system process (Supplementary Table 3). Additionally, an overall enrichment was found for genes associated with developmental processes. These findings suggest that altered methylation status may disrupt the expression of neurodevelopmental genes and may play a role in the pathophysiology of FHS.

## Discussion

The identification of a unique methylation profile associated with FHS suggests that truncating mutations of SRCAP, the genetic cause underlying FHS, result in recurrent, locus specific, DNA methylation alterations. These may be the direct result of altered function of the truncated SRCAP protein; alternatively, they may be the result of a secondary compensatory mechanism in response to altered SRCAP function. Regardless of the underlying molecular mechanism, these findings suggest a role for SRCAP in the regulation of genomic DNA methylation, which in turn may regulate the expression of specific genes.

Rare genetic diseases caused by mutations in genes involved in the regulation of methylation have been recognized for more than 15 years. For example, mutations in the epigenetic regulatory gene *ATRX* were shown to cause X-linked mental retardation with α-thalassemia (ATRX syndrome) in ref. 24 and, in early studies, disease-causing mutations were shown to alter the methylation patterns of several repetitive genomic regions including Y-specific satellite and subtelomeric repeats^22^. Mutations in the histone H3 lysine 4 demethylase, KDM5C, were identified to cause an X-linked form of intellectual disability syndrome in ref. 25. More recently, patients with mutations in KDM5C were studied using a DNA methylation array approach, providing evidence of recurrent global DNA methylation defects in the peripheral blood as well as post-mortem brain tissue samples of these individuals^26^. Most recently, a unique methylation episignature was reported for Sotos syndrome, a rare overgrowth disorder caused by mutations in the *NSD1* gene, encoding histone H3 lysine 36 methyltransferase^27^. This study utilized the same high resolution methylation array used here and was able to distinguish individuals with Sotos syndrome (secondary to pathogenic NSD1 mutations) from individuals with non-pathogenic mutations of NSD1, as well as from cases of the clinically similar disorder Weaver syndrome, caused by mutations in the histone methyltransferase *EZH2* (Enhancer of Zeste, Drospholia, Homolog 2). This field is currently in its infancy and we anticipate that similar genome-wide epi-signatures will be characterized for this emerging group of rare diseases in the years to come.

How the epigenetic consequences of these disease-causing mutations actually result in the rare disease itself is not well understood. It may be anticipated that the methylation alterations could result in differences in transcriptional regulation. For example, hypomethylation in a gene promoter CpG island may result in increased transcription, whereas hypermethylation may result in decreased transcriptional activity. Our pathway analysis for FHS, and that recently reported for Sotos syndrome^27^, suggest impact on genes that might be relevant to the cardinal developmental processes disrupted in these rare diseases; however, further research is necessary to fully understand the downstream consequences of these methylation alterations, particularly given that their impact is predicted to be cell-, tissue- and developmental timing- specific. Such data will provide new insight into the pathogenesis of these developmental disorders.

Here we presented a pipeline to sensitively and specifically detect regional methylation differences in a cohort of patients. Our analysis employs a region detection algorithm that controls for the most common limitations of the array. Probes containing SNPs may affect the assessment of DNA methylation when analyzing single-probe methylation, as described in the study by Price *et al*.^28^. One way to address this is to employ a region detection algorithm, which relies in multiple adjacent probes (at least 3) to meet significant stringent p-value, F-value and mean methylation cut off criteria. A single polymorphic probe would not be expected to significantly affect the methylation results across a multi-probe region. Furthermore, the difference in performance of Infinium I and Infinium II was demonstrated to be minimum and so do not significantly affect differential methylation detection^29^. The authors found an average shift on beta values of 2 to 8% on Infinium II assay probe sets. In fact, similarly to the SNP related effects, a region detection algorithm is used in part to address this feature. Considering that our analysis include a cut off of at least 15% methylation difference, and employs a multi-probe region detection algorithm, the effect of the probe chemistry is expected to be negligible. Age-related and cell composition-related changes in the DNA methylation have been observed including our recent description of global DNA methylation changes with age using the Illumina 450 K array^30^. Some possible explanations for this include differences in composition of nucleated cell subtypes in premature births versus newborns and children (ie. increased levels of nucleated red blood cells; differences in the levels of leukocyte subtypes), or alternatively global methylation differences associated with maturation of leukocytes. For this reason our methylation analysis includes a large reference cohort with a broad range of ages (from 1 month to 62 years). This allows the exclusion of sites with methylation changes related to age, and consequently cell type, as such genomic regions present with hyper variable DNA methylation profiles in the reference cohort, and would not meet the statistical cut off criteria. Finally, the ability of our algorithm to sensitively and specifically detect methylation differences across this patient cohort was demonstrated by a confirmatory analysis on a subset of these regions using the “gold standard” Clonal Bisulfite Sequencing.

In conclusion, our findings demonstrate the existence of a unique DNA methylation epi-signature in the peripheral blood of individuals with FHS. The epi-signature provides further insight into the FHS disease etiology, and represents a potential diagnostic biomarker for this disorder. Identification of similar types of epi-signatures in constitutional genetic syndromes will further expand our understanding of these diseases and facilitate the efficient diagnosis of these individuals.

## Methods

### Methylation Array and Analysis

Global methylation status of 18 individuals with mutation-confirmed FHS was performed using the Infinium HumanMethylation450 Beadchip (Illumina) methylation arrays at the Genetic and Molecular Epidemiology Laboratory at McMaster University, according to manufacturer’s instructions. Blood-derived DNA samples were obtained from 9 FHS individuals with the most common FHS-causing *SRCAP*mutation (c.7330C > T; p.Arg2444*) and 9 additional FHS individuals with distinct mutations in SRCAP (c.7165G > T; p.Glu2389*, c.7218_7219delTC; p.Gln2407fs*35, c.7219C > T; p.Gln2407*, c.7282dupC; p.Arg2428fs*15, c.7303C > T; p.Arg2435*, c.7316dupC; p.Ala2440fs*3, c.7549delC; p.Gln2517fs*5, c.8117C > G; p.Ser2706*, and c.8242C > T; p.Arg2748*). FHS cohort included 6 males and 12 females with mixed ethnicity, which ranged in age from 2 to 42 years (Supplementary Fig. 4). The FHS individual cohort was compared to an unmatched reference control cohort of 361 individuals (151 females and 210 males) of mixed ethnicity, with average age of 8.5 years (0–62 years; Supplementary Fig. 4). Our reference cohort included individuals that were previously preselected from a larger cohort of about 1000 individuals across the broad range of age, sex and ethnicity distribution. The methylation analysis of these individuals was performed in the same facility as patients and same data processing pipeline was used. Based on the individual analysis (1 sample vs cohort) these reference controls showed no significant changes in DNA methylation relative to the entire reference cohort. This analysis takes into account the fact that significant portion of genomic DNA methylation is hyper-variable across individuals (including age-related hyper-variable regions). Such regions with the normal inter-individual and/or age-related methylation variability would not produce significant p-values when comparing an individual or a patient cohort to a reference. Therefore, this analytical approach is designed to take into consideration methylation variability including sex and age, while taking advantage of analytical power of a large reference control database and focuses on identification of unique, non-age/sex variable DNA methylation changes in individual patients.

Methylation array coverage included >485,000 individual methylation sites, 99% of RefSeq genes and 96% of annotated CpG islands. Methylation values were generated using the Illumina Genome Studio Software, and data (.idat files) containing β-values were imported in the Partek Genomic Suite (PGS) software. Data (.idat files) were normalized using the PGS Genome Studio Normalization Algorithm that includes background subtraction and control normalization (normalized value = original value * target mean/control mean), while removing individual probes with poor signal intensity. An ANOVA test was performed to determine the methylation estimate (net methylation difference in FHS individuals as compared to controls) and generate probe-level statistics, including p-value (t-test), and F value (signal to noise ratio). Genomic regions with significant DNA methylation patterns were identified that met the following statistical criteria: (1) minimum of 3 consecutive probes with significant methylation change p < 0.01; (2) mean F-value across the region >50; and, (3) methylation estimate value differing by more than 20%. Probes on the X chromosome were further analyzed by comparing sex matched FHS individuals and controls. Significant regions were mapped against the CpG islands and gene promoter regions using Hg19 as reference genome. Data was visualized using PGS genomic browser. Lastly, regions with the most significant methylation changes were annotated in reference to the location of the CpG islands and distance to gene promoters. This analytical methods have been developed and validated previously^31^,^32^ and were performed in accordance with the relevant REB guidelines and regulations.

### Age and cell composition prediction

DNA methylation age and blood cell composition measures derived from the DNA methylation data were performed using the Epigenetic Clock software developed by Horvath, 2013^33^. Briefly DNA methylation age, defined as predicted age, was calculated based on 21,369 CpG probes that were present both on the Illumina 450 K and 27 K platform and had fewer than 10 missing values. Age acceleration was measured by the difference between predicted age and chronological age. Blood cell proportions of CD8 T cells, CD4 T cells, natural killer cells, B cells, monocytes and granulocytes were estimated as described by Houseman *et al*.^34^.

### Clonal Bisulfite Sequencing and Analysis

Blood-derived DNA samples from four FHS probands (three from the original array and an independent FHS sample) and two control subjects were bisulfite converted using the EZ DNA Methylation-Direct Kit according to manufacturer’s instructions (Zymo Research). Methylation-specific primers were designed for each region using the software program MethPrimer^35^. Converted DNA was PCR amplified using methylation primer pairs specific to each gene region and the resulting PCR amplicons were subcloned into the pGEM-T Easy Vector (Promega). For *MYO1F*, a nested-primer PCR strategy was used for amplification. Recombinant vectors were transformed into Top10 chemically competent *E. coli* cells (ThermoFisher). Transformant colonies were picked for clonal PCR amplification and subsequently Sanger sequenced. Chromatogram sequencing results for ≥20 clones were manually analyzed and string diagrams were generated for each affected individual and region.

### Methylation Array Pathway Analysis

The 116 differentially methylated genes identified by methylation array (Supplementary Table 1) were assessed using the pathway analysis tool in the Partek Genomics Suite software. Briefly, statistical analysis included Fisher Exact test and Chi Square test, and was restricted to functional groups at least two genes. Results show the Enrichment p-value (*p*-value of the Fisher Exact test and Chi Square test reflective of the number of the genes in vs. not in the list or functional group) and the Enrichment score (negative log of the enrichment *p*-value; a high score indicates that the genes in the functional group are overrepresented in the gene list).

### Ethical approval and consent to participate

The CHEO Research Ethics Board approved the project. All analytical methods were performed in accordance with the relevant REB guidelines and regulations. All patients provided a written informed consent before their inclusion in the study, in accordance with the Declaration of Helsinki.

## Additional Information

**How to cite this article**: Hood, R. L. *et al*. The defining DNA methylation signature of Floating-Harbor syndrome. *Sci. Rep.* **6**, 38803; doi: 10.1038/srep38803 (2016).

**Publisher's note:** Springer Nature remains neutral with regard to jurisdictional claims in published maps and institutional affiliations.

## Supplementary Material

Supplementary Information

## Figures and Tables

**Table 1:** Regions with significantly altered methylation (>20%) in FHS individuals identified by methylation array.

Location	Region Start^a^	Region Stop^a^	Region Length (bp)	# Probes	Methylation Estimate^b^	Nearest Gene	Overlapping CpG Island
chr1	174843744	174843981	238	3	0.2428	RABGAP1L (+)	No
chr1	27676195	27676662	468	3	−0.2197	SYTL1 (+)	Yes
chr1	1003116	1003539	424	4	−0.2773	RNF223 (−)	Yes
chr2	164204618	164205353	736	7	0.3223	FIGN (−)^**^	Yes
chr3	159557542	159558041	500	4	0.2235	SCHIP1 (+)	No
chr4	99064092	99064914	823	9	0.2394	STPG2 (−)^**^	Yes
chr4	46126056	46126458	403	7	0.2392	GABRG1 (−)	No
chr4	62382922	62383250	329	4	0.2065	LPHN3 (+)	Yes
chr4	11370304	11370882	579	5	0.2028	MIR572 (+)	Yes
chr5	110062333	110062847	515	7	0.2514	TMEM232 (−)	No
chr5	42944020	42944504	485	4	0.2232	FLJ32255 (−)	Yes
chr7	32358054	32358550	497	3	0.2215	LOC100130673 (−)	No
chr7	92672802	92673186	385	5	0.2094	SAMD9 (−)	Yes
chr8	81478162	81478344	183	3	0.2572	ZBTB10 (+)	No
chr8	39172010	39172130	121	6	0.2537	ADAM5 (+)	No
chr8	102235917	102236841	925	6	0.2057	ZNF706 (−)	Yes
chr9	139258514	139259084	571	3	−0.2055	CARD9 (−)	Yes
chr10	89167447	89167981	535	4	0.2216	LINC00864 (−)	No
chr10	50649656	50650258	603	5	0.2001	ERCC6 (−)	No
chr12	75784531	75785305	775	11	0.2007	GLIPR1L2 (+)	Yes
chr13	23412240	23412632	393	4	0.2263	BASP1P1 (−)	Yes
chr19	49222477	49224464	1988	12	−0.2017	RASIP1 (−)^**^	Yes
chr19	1063614	1064228	615	3	−0.2126	ABCA7 (+)	Yes
chr19	523290	523652	363	3	−0.2162	TPGS1 (+)	Yes
chr19	49133411	49133855	445	4	−0.2469	DBP (−)	Yes
chr19	8591354	8591786	433	4	−0.3725	MYO1F (−)^**^	Yes
chr20	62679245	62679723	479	3	0.2034	SOX18 (−)	Yes
chr22	50737968	50738900	933	4	−0.254	PLXNB2 (−)	Yes

Significantly methylated regions met the following criteria: Estimate value >20%, F value>50, and p < 0.01.

a. hg19 Location; bp.

b. Positive methylation estimate values indicate hypermethylation whereas negative values indicate hypomethylation in FHS subjects compared to controls.

**Indicates regions used for bisulfite sequencing confirmation analysis.

Abbreviations: chr = chromosome; bp = base pair; (+) = sense strand; (−) = anti-sense strand.

**Table 2:** Genomic region distribution of the 28 differentially methylated regions (>20%) in FHS individuals.

	Within CpG island	Outside CpG island
Within gene		
Gene body	9	1
Promoter	3	4
Intergenic	7	4

No regions detected in CpG shores and shelves.
