# Supplementary material for: The defining DNA methylation signature of Floating-Harbor Syndrome
Source: Sci Rep. 2016 Dec 9;6:38803. doi: 10.1038/srep38803 (PMC5146968; doi:10.1038/srep38803)

Supplementary Information 1

The defining DNA methylation signature of Floating-Harbor Syndrome

Rebecca L. Hood, Laila C. Schenkel, Sarah M. Nikkel, Peter J. Ainsworth, Guillaume Pare, Kym M. Boycott, Dennis E. Bulman, and Bekim Sadikovic

Supplementary Table 1. Regions with significantly altered methylation (>15%) in FHS individuals identified by methylation array

Location	Region Start (hg19 location; bp)	Region Stop (hg19 location; bp)	Region Length (bp)	Number of Probes in Region	Methylation Estimate[a]	p-value[b]	Nearest gene	Overlapping CpG Island
chr1	1003116	1003539	424	4	-0.27727	4.46E-05	RNF223 (-)	Yes
chr1	45278961	45279694	734	6	-0.15405	4.46E-05	BTBD19 (+)	Yes
chr1	174843744	174843981	238	3	0.242846	4.46E-05	RABGAP1L (+)	No
chr1	169429594	169429982	389	3	0.193606	4.46E-05	CCDC181 (-)	No
chr1	224363439	224363585	147	3	0.160763	0.00766933	DEGS1 (+)	No
chr1	95698817	95699323	507	6	0.151871	4.46E-05	LOC101928118 (-)	No
chr1	2979301	2980947	1647	10	-0.18146	4.46E-05	LINC00982 (-)	Yes
chr1	27676195	27676662	468	3	-0.21966	0.0061533	SYTL1 (+)	Yes
chr1	178455597	178456280	684	5	0.166236	4.46E-05	RASAL2 (+)	Yes
chr1	211652266	211652751	486	4	0.152705	4.46E-05	RD3 (-)	Yes
chr1	143663852	143664141	290	3	0.173754	4.46E-05	MIR6077 (+)	Yes
chr1	228890791	228891316	526	6	0.160177	0.00124849	RHOU (+)	Yes
chr1	43814159	43815475	1317	7	-0.17638	4.46E-05	MPL (+)	Yes
chr1	1229095	1229800	706	6	-0.16002	0.000178356	ACAP3 (-)	Yes
chr1	108022757	108023492	736	6	0.155427	0.00209569	NTNG1 (+)	Yes
chr1	2120975	2121734	760	6	-0.19186	4.46E-05	C1orf86 (-)	Yes
chr1	149162209	149162528	320	4	0.154546	4.46E-05	LOC388692 (+)	Yes
chr1	38461530	38461906	377	4	-0.17179	4.46E-05	FHL3 (-)	Yes
chr1	55246857	55247418	562	5	0.154141	0.00133767	TTC22 (-)	Yes
chr2	164204618	164205353	736	7	0.322347	4.46E-05	FIGN (-)	Yes
chr2	50201372	50201521	150	5	0.189996	4.46E-05	NRXN1 (-)	No
chr2	173539081	173539631	551	4	0.158672	4.46E-05	RAPGEF4-AS1 (-)	No
chr2	105735607	105735766	160	3	0.171228	4.46E-05	LOC101927492 (-)	No
chr2	129659306	129659956	651	6	0.196434	4.46E-05	LOC101927881 (+)	Yes
chr2	87036616	87037048	433	4	0.190747	4.46E-05	CD8B (-)	Yes
chr2	96191005	96191364	360	3	0.163192	0.000312124	TRIM43B (-)	Yes
chr3	87137923	87138710	788	7	0.168147	4.46E-05	LINC00506 (+)	Yes
chr3	109056339	109056907	569	4	0.155988	4.46E-05	DPPA4 (-)	No
chr3	159557542	159558041	500	4	0.223463	0.000445891	SCHIP1 (+)	No
chr3	156323942	156324128	187	3	0.152503	4.46E-05	SSR3 (-)	No
chr3	113160061	113160647	587	9	0.16965	4.46E-05	CFAP44 (-)	Yes
chr3	105072527	105073087	561	3	0.196409	4.46E-05	ALCAM (+)	Yes
chr3	350493	351013	521	6	0.173252	4.46E-05	CHL1 (+)	Yes
chr4	99064092	99064914	823	9	0.239439	4.46E-05	STPG2 (-)	Yes
chr4	46126056	46126458	403	7	0.239231	4.46E-05	GABRG1 (-)	No

chr4	165898656	165898977	322	8	0.193923	4.46E-05	TRIM61 (-)	No
chr4	1512860	1513269	410	5	-0.16639	4.46E-05	NKX1-1 (-)	No
chr4	62382922	62383250	329	4	0.206485	4.46E-05	LPHN3 (+)	Yes
chr4	14864533	14864873	341	3	0.151104	4.46E-05	CPEB2-AS1 (-)	Yes
chr4	11370304	11370882	579	5	0.202768	4.46E-05	MIR572 (+)	Yes
chr4	155702399	155703148	750	6	0.191892	4.46E-05	RBM46 (+)	Yes
chr5	11588961	11589059	99	3	0.18154	4.46E-05	CTNND2 (-)	No
chr5	42756776	42757181	406	5	0.189881	4.46E-05	CCDC152 (+)	No
chr5	78985415	78985910	496	10	0.154164	4.46E-05	CMYA5 (+)	No
chr5	110062333	110062847	515	7	0.251366	4.46E-05	TMEM232 (-)	No
chr5	42944020	42944504	485	4	0.2232	4.46E-05	FLJ32255 (-)	Yes
chr5	145758576	145758891	316	5	0.15963	4.46E-05	POU4F3 (+)	Yes
chr5	78365245	78366086	842	7	0.162276	4.46E-05	BHMT2 (+)	Yes
chr5	8457538	8458402	865	7	0.171172	0.000668837	MIR4458HG (+)	Yes
chr6	49681168	49681784	617	9	0.174187	4.46E-05	CRISP2 (-)	No
chr6	17016216	17016494	279	3	0.159429	4.46E-05	STMND1 (+)	No
chr6	32847367	32847855	489	22	0.178924	0.000668837	LOC100294145 (+)	Yes
chr7	39170487	39171123	637	6	0.186442	4.46E-05	POU6F2 (+)	No
chr7	32358054	32358550	497	3	0.221514	4.46E-05	LOC100130673 (-)	No
chr7	143582136	143582640	505	4	0.176392	4.46E-05	FAM115A (-)	Yes
chr7	92672802	92673186	385	5	0.209416	4.46E-05	SAMD9 (-)	Yes
chr7	16890421	16891089	669	6	0.151387	0.000222946	AGR3 (-)	Yes
chr8	81478162	81478344	183	3	0.2572	4.46E-05	ZBTB10 (+)	No
chr8	39172010	39172130	121	6	0.253711	4.46E-05	ADAM5 (+)	No
chr8	2585656	2586235	580	3	0.18257	0.00111473	LOC101927815 (-)	No
chr8	74282802	74282941	140	3	0.194527	4.46E-05	LOC101926926 (-)	No
chr8	119086570	119086772	203	3	0.164421	0.000133767	EXT1 (-)	No
chr8	54605556	54605798	243	3	0.161386	4.46E-05	ATP6V1H (-)	No
chr8	145730808	145731419	612	3	-0.1794	0.000178356	GPT (+)	Yes
chr8	102235917	102236841	925	6	0.20572	4.46E-05	ZNF706 (-)	Yes
chr8	99984524	99985059	536	4	0.172668	0.00552905	OSR2 (+)	Yes
chr8	67454536	67454902	367	5	0.191337	4.46E-05	MYBL1 (-)	Yes
chr9	139258514	139259084	571	3	-0.20546	4.46E-05	CARD9 (-)	Yes
chr10	89167447	89167981	535	4	0.221604	4.46E-05	LINC00864 (-)	No
chr10	50649656	50650258	603	5	0.200051	0.000445891	ERCC6 (-)	No
chr10	81743118	81743556	439	4	0.157609	4.46E-05	SFTPD (-)	Yes
chr11	106698552	106698704	153	3	0.154062	0.000445891	GUCY1A2 (-)	No
chr11	85393683	85393905	223	3	0.153076	0.00222946	CCDC89 (-)	No
chr11	65360113	65360519	407	5	-0.1999	4.46E-05	KCNK7 (-)	Yes
chr11	58830181	58830867	687	5	0.151247	4.46E-05	FAM111B (+)	Yes
chr12	75784531	75785305	775	11	0.200664	4.46E-05	GLIPR1L2 (+)	Yes

chr12	21926437	21926534	98	3	0.151756	0.00120391	KCNJ8 (-)	No
chr12	95840303	95840905	603	5	0.176415	4.46E-05	METAP2 (+)	No
chr12	58011754	58011885	132	4	0.167664	4.46E-05	LOC101927583 (-)	No
chr12	64215601	64215907	307	3	0.188272	0.000445891	TMEM5 (+)	Yes
chr13	23309764	23310685	922	9	0.184989	4.46E-05	BASP1P1 (-)	No
chr13	31506675	31507149	475	8	0.16312	4.46E-05	TEX26 (+)	No
chr13	23412240	23412632	393	4	0.226345	4.46E-05	BASP1P1 (-)	Yes
chr13	23270675	23270860	186	3	0.158339	4.46E-05	BASP1P1 (-)	Yes
chr13	110521946	110522307	362	5	0.15779	0.000401302	IRS2 (-)	Yes
chr15	23157717	23158348	632	4	0.161761	4.46E-05	WHAMMP3 (-)	Yes
chr15	40583217	40583432	216	3	-0.18611	4.46E-05	PLCB2 (-)	Yes
chr15	69222582	69223378	797	4	0.151475	4.46E-05	SPESP1 (+)	Yes
chr15	29034659	29034960	302	3	0.158286	0.000178356	PDCD6IPP2 (+)	Yes
chr16	30907236	30907689	454	3	-0.15839	4.46E-05	CTF1 (+)	No
chr16	279736	280056	321	3	-0.19244	4.46E-05	LUC7L (-)	No
chr16	57562442	57563405	964	5	-0.15829	0.000178356	CCDC102A (-)	Yes
chr17	33842171	33842311	141	3	0.151089	4.46E-05	SLFN12L (-)	No
chr17	9550127	9550555	429	5	0.158825	0.00120391	USP43 (+)	No
chr17	17603521	17604194	674	6	-0.16929	4.46E-05	RAI1 (+)	Yes
chr17	40835839	40836135	297	4	0.173002	4.46E-05	CNTNAP1 (+)	Yes
chr17	46641494	46642114	621	5	-0.15273	4.46E-05	HOXB3 (-)	Yes
chr18	32173074	32173237	164	4	0.171691	4.46E-05	DTNA (+)	No
chr18	19476854	19477069	216	4	0.196499	4.46E-05	MIR1-2 (-)	Yes
chr19	49222477	49224464	1988	12	-0.20169	4.46E-05	RASIP1 (-)	Yes
chr19	8591354	8591786	433	4	-0.37247	4.46E-05	MYO1F (-)	Yes
chr19	1063614	1064228	615	3	-0.21259	4.46E-05	ABCA7 (+)	Yes
chr19	18543819	18544429	611	3	-0.17983	4.46E-05	SSBP4 (+)	Yes
chr19	3480353	3480682	330	5	-0.17453	0.00931912	SMIM24 (-)	No
chr19	46801547	46801682	136	3	-0.15352	4.46E-05	HIF3A (+)	No
chr19	2428112	2429219	1108	7	-0.15526	4.46E-05	LMNB2 (-)	Yes
chr19	49133411	49133855	445	4	-0.24687	4.46E-05	DBP (-)	Yes
chr19	523290	523652	363	3	-0.21615	0.000312124	TPGS1 (+)	Yes
chr20	62693647	62694015	369	7	-0.16163	4.46E-05	TCEA2 (+)	Yes
chr20	62679245	62679723	479	3	0.203354	4.46E-05	SOX18 (-)	Yes
chr22	50737968	50738900	933	4	-0.25404	4.46E-05	PLXNB2 (-)	Yes
chrX	111623890	111624333	444	4	0.173589	4.46E-05	ZCCHC16 (+)	Yes
chrX	11157132	11157620	489	5	0.184296	0.00129308	ARHGAP6 (-)	Yes
chrY	6114245	6114414	170	3	-0.1983	0.000178356	TSPY2 (+)	Yes
chrY	9385529	9385871	343	3	-0.18799	0.00374549	TSPY10 (+)	Yes
chrY	21239338	21240014	677	4	0.162264	0.00124849	CD24 (-)	Yes

Significantly altered regions listed in this table have: methylation estimate value >15%, F value>50, and p<0.01.

a. Positive Estimate values indicate hypermethylation and negative Estimate values indicate hypomethylation in FHS subjects compared to controls.

b. p-value shows the significance for the region (within minimum of 3 probes)

Abbreviations: chr = chromosome; bp = base pair; (+) = sense strand; (-) = anti-sense strand

Supplementary Table 2. Genomic region distribution of the 116 differentially methylated regions (>15%) in FHS individuals.

	Within CpG island	Within CpG shores	Within CpG shelves	Outside CpG site
**Within gene body**	42	4	0	20
**Outside gene body**	31	4	0	19

Supplementary Table 3. Pathway analysis of the differentially methylated genes in FHS.

**Biological Pathway**	Neurological System Process	Synaptic Transmission	Developmental Process
**Number of genes in group**	10	8	29
**Fisher Exact Enrichment Score -ln(p-value)**	4.967	7.048	3.973
**Fisher Exact right-tail p-value**	0.0069	0.00087	0.0188
**Chi Square Enrichment Score -ln(p-value)**	6.06099	10.9996	3.7652
**Chi Square p-value**	0.00233	1.67084e-005	0.02316
**Genes**	ABCA7<br>CHL1<br>CNTNAP1<br>CTNND2<br>NKX1-1<br>NRXN1<br>PLCB2<br>POU4F3<br>POU6F2<br>RD3	CD24<br>DTNA<br>GABRG1<br>KCNJ8<br>KCNK7<br>NRXN1<br>PLCB2<br>TPGS1	CD24<br>CHL1<br>CNTNAP1<br>CTF1<br>CTNND2<br>DBP<br>DPPA4<br>ERCC6<br>EXT1<br>FHL3<br>HIF3A<br>HOXB3<br>IRS2<br>KCNJ8<br>NRXN1<br>NTNG1<br>OSR2<br>PLXNB2<br>POU4F3<br>POU6F2<br>RAI1<br>RASIP1<br>RD3<br>SFTPD<br>SOX18<br>SPESP1<br>TPGS1<br>TSPY2<br>TSPY10

Supplemental Figure Legends:

Supplementary Figure 1. Histogram comparison of differential FHS methylation estimates (a) within CpG islands and (b) outside of CpG islands.

The frequency of individual probes with significant methylation differences (Y-axis) with methylation estimates >20% (X-axis) in FHS individuals compared to controls. Positive estimate values show hypermethylation in FHS, negative values show hypomethylation in FHS. (a) Methylation of statistically significant regions within CpG Islands. (b) Methylation of statistically significant regions outside CpG Islands.

Supplementary Figure 2. Blood cell measures. Estimated proportions of CD8 T cells (CD8T), CD4T cells (CD4T), natural killer cells (NK), B cells, monocytes (Mono) and granulocytes (Gran) in FHS patients and controls based on Illumina 450K methylation data.

Supplementary Figure 3. Methylation of significantly altered regions in FHS individuals compared to controls based on methylation array data. Methylation array data: level from 0 (not methylated) to 1 (100% methylated) is plotted for individual FHS individual and control samples for regions with significantly altered methylation in FHS: hypermethylated regions (*MYO1F* and *RASIP1*) and hypomethylated regions (*FIGN* and *STPG2*). Each individual in the control (red) and FHS individual (blue) groups are represented as a circle. Corresponding box and whisker plots illustrate the methylation median, upper and lower quartiles, and standard deviation for FHS individuals compared to controls.

Supplementary Figure 4. Age distribution of (A) 361 controls and (B) 18 patients with FHS included in this study. X axis shows the age in years and Y axis shows the frequency observed.

The defining DNA methylation signature of Floating-Harbor Syndrome

Rebecca L. Hood, Laila C. Schenkel, Sarah M. Nikkel, Peter J. Ainsworth, Guillaume Pare, Kym M. Boycott, Dennis E. Bulman, and Bekim Sadikovic

Supplementary Figure 1:

A — Regions within CpG Islands

B — Regions outside CpG Islands

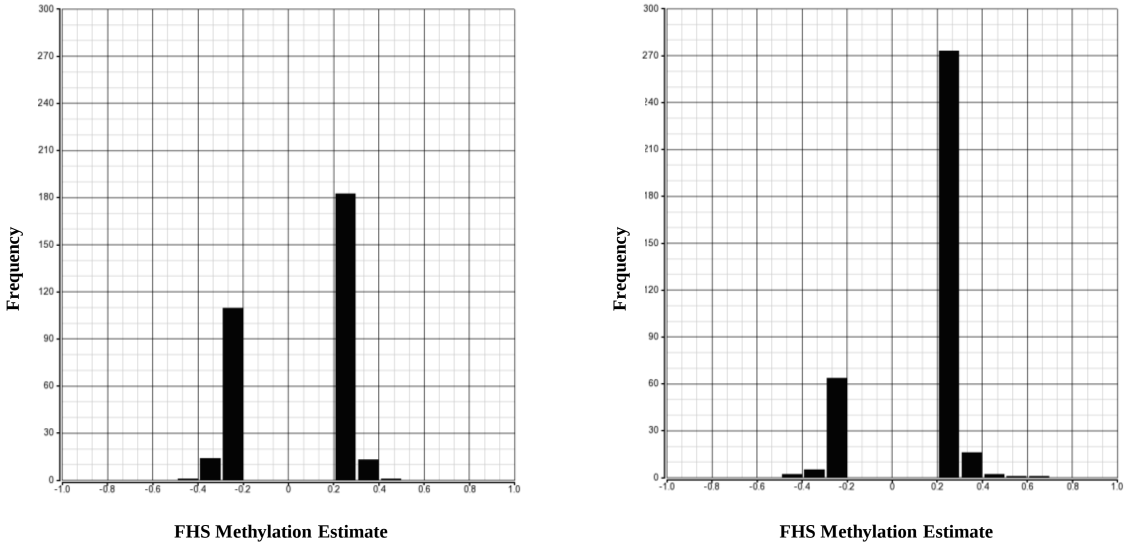

The defining DNA methylation signature of Floating-Harbor Syndrome

Rebecca L. Hood, Laila C. Schenkel, Sarah M. Nikkel, Peter J. Ainsworth, Guillaume Pare, Kym M. Boycott, Dennis E. Bulman, and Bekim Sadikovic

Supplementary Figure 2:

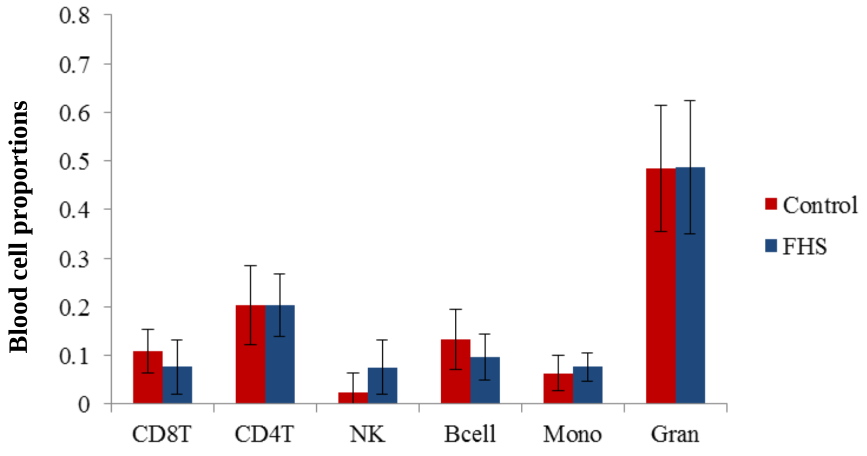

The defining DNA methylation signature of Floating-Harbor Syndrome

Rebecca L. Hood, Laila C. Schenkel, Sarah M. Nikkel, Peter J. Ainsworth, Guillaume Pare, Kym M. Boycott, Dennis E. Bulman, and Bekim Sadikovic

Supplementary Figure 3:

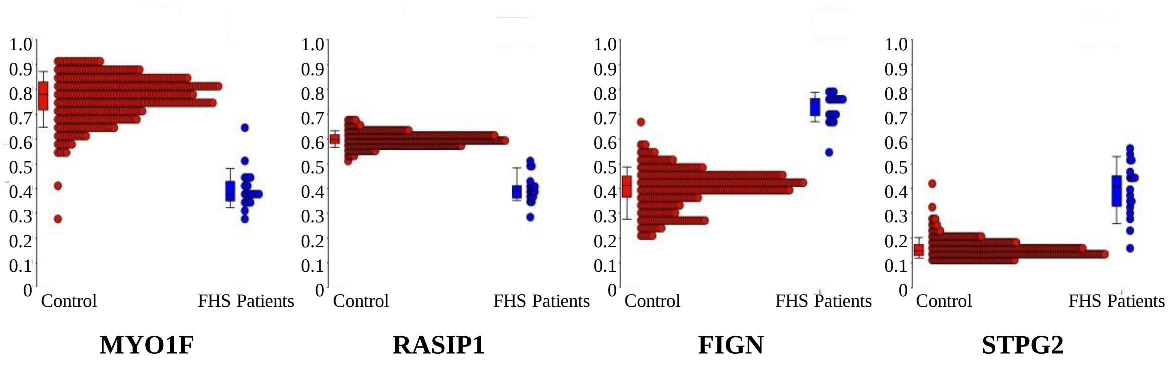

The defining DNA methylation signature of Floating-Harbor Syndrome

Rebecca L. Hood, Laila C. Schenkel, Sarah M. Nikkel, Peter J. Ainsworth, Guillaume Pare, Kym M. Boycott, Dennis E. Bulman, and Bekim Sadikovic

Supplementary Figure 4:

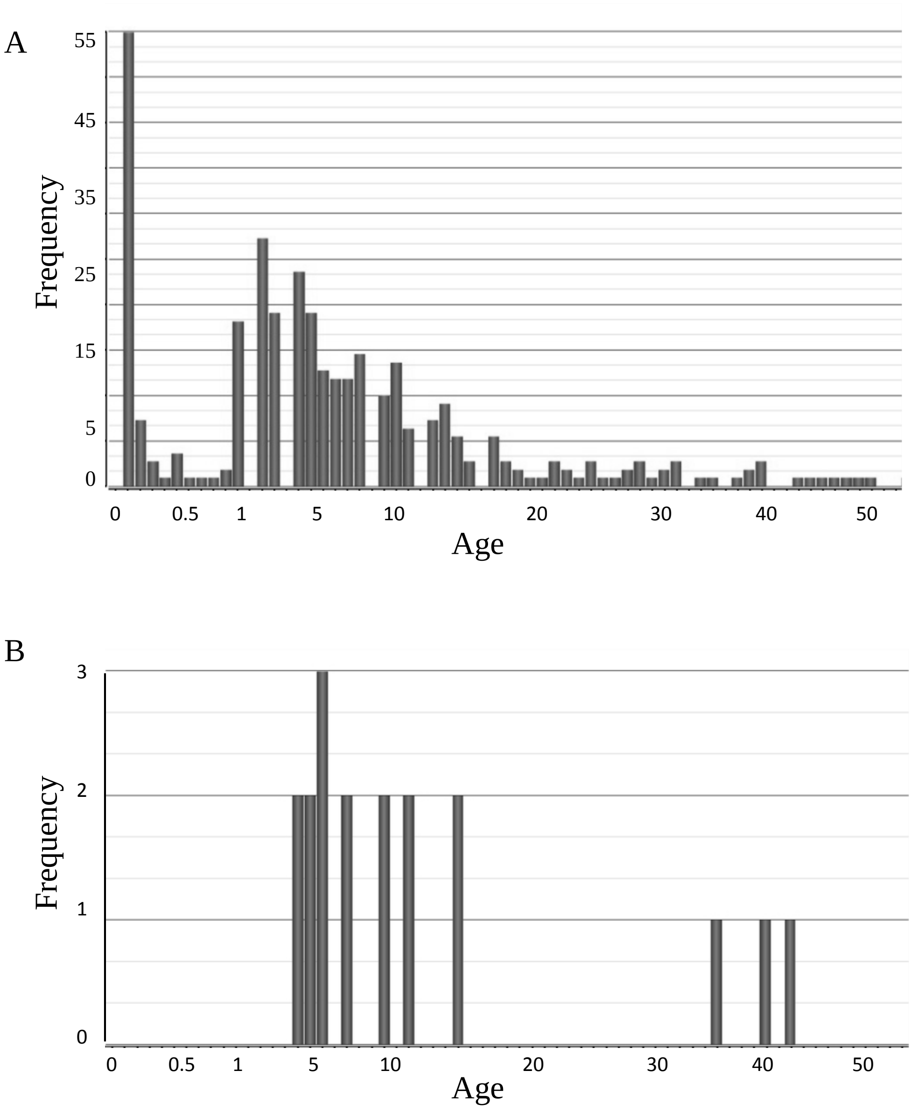

Supplement: Supplementary Information [file srep38803-s1.pdf]
